# Facial spasm and headaches: should we call it “IIH-Spasm syndrome?”

**DOI:** 10.1186/1129-2377-1-S14-P225

**Published:** 2013-02-21

**Authors:** S Muzerengi, C Moor, M Davies

**Affiliations:** 1 University Hospital of North Staffordshire NHS trust, UK

## Introduction

Idiopathic intracranial hypertension (IIH) typically manifests with headaches and visual disturbance. Unusual presenting features of IIH are sometimes described. Hemi facial spasm (HFS) in women has an incidence of 0.81 per 100,000 compared to an IIH incidence rate of 3.5-20 per 100,000. We describe the 4th case of HFS induced by IIH and we propose a new nomenclature.

## Case

We present a case of a 33 year old right handed woman with sequential new onset headache causing secondary HFS. Neurological examination revealed bilateral papillaoedema and confirmed right sided facial spasms. Cerebrospinal fluid examination showed an opening pressure of 33cm H2O and normal CSF constituents. High resolution brain MRI and MRV were normal including no evidence of neurovascular compression in the cerebello-pontine angle. EEG was entirely normal. The headache and HFS resolved after lowering CSF pressure. At three months post lumbar puncture she remained asymptomatic with no further episodes of HFS. Conclusion: There are only three prior cases of IIH associated with HFS in the published literature. We describe a further case of a very rare clinical manifestation of IIH and propose a possible pathophysiological mechanism and a new name for this entity “IIH-Spasm syndrome”.
